# Associations between social and built environments and cardiovascular disease mortality across 342 Latin American cities: results from the SALURBAL study

**DOI:** 10.7189/jogh.15.04262

**Published:** 2025-10-31

**Authors:** Dayan Carvalho Ramos Salles de Oliveira, Nelson Gouveia, Natalia Tumas, Carolina Nazzal, Tania Alfaro, Maria Fernanda Kroker-Lobos, Jaime Miranda, Manuel Ramirez-Zea, Thais Oliveira, Waleska Caiaffa, Goro Yamada, Usama Bilal, Letícia de Oliveira Cardoso

**Affiliations:** 1 National School of Public Health, Oswaldo Cruz Foundation, Rio de Janeiro, Brazil; 2 Department of Preventive Medicine, University of Sao Paulo Medical School, Sao Paulo, Brazil; 3 Centro de Investigaciones y Estudios sobre Cultura y Sociedad, Consejo Nacional de Investigaciones Científicas y Técnicas (CONICET) y Universidad Nacional de Córdoba, Argentina; 4 Facultad de Ciencias Médicas, Universidad Nacional de Córdoba, Córdoba, Argentina; 5 Johns Hopkins University, Universitat Pompeu Fabra Public Policy (JHU-UPF PPC), Universitat Pompeu Fabra (UPF), UPF Barcelona School of Management (UPF-BSM), Barcelona, Spain; 6 School of Public Health, University of Chile, Santiago, Chile; 7 Institute of Nutrition of Central America and Panama, INCAP Research Center for the Prevention of Chronic Diseases, Guatemala City, Guatemala; 8 Universidad Peruana Cayetano Heredia, CRONICAS Center of Excellence in Chronic Diseases, Lima, Peru; 9 Observatory for Urban Health in Belo Horizonte, Federal University of Minas Gerais, Minas Gerais, Brazil; 10 Drexel University, Dornsife School of Public Health, Philadelphia, USA

## Abstract

**Background:**

Latin American cities have heterogeneous mortality and urban environment profiles. We aim to describe the variability in cardiovascular disease (CVD) mortality across and within cities, and within and between Latin American countries, and investigate the associations between urban environmental features and CVD mortality in these areas.

**Methods:**

Using harmonised data from the *Salud Urbana en América Latina* (*i.e.* Urban Health in Latin America) project, we investigated associations between urban features and CVD mortality in 342 Latin American cities encompassing 1252 sub-city units in nine Latin American countries. We obtained CVD mortality data from vital statistics and population denominators from national census bureaus. We evaluated the social environment through an educational attainment index from national censuses. Built environment indicators covered urban form, landscape, street design, transportation, and greenness. We applied multilevel negative binomial models with random intercepts for sub-city units nested within cities to describe the associations of social and built environmental features with CVD mortality adjusted by age and sex in each country.

**Results:**

CVD death rates varied across sub-cities within and between Latin American countries, with greater variability in age-adjusted CVD death rates between sub-cities within cities (60%) than between cities within countries (21%) and between countries (19%). In fully adjusted models, higher educational attainment in sub-cities (rate ratio (RR) = 0.95; 95% confidence interval (CI) = 0.94–0.96) and higher landscape fragmentation in cities (RR = 0.97; 95% CI = 0.95–0.99) were associated with lower CVD mortality. Conversely, higher street connectivity in sub-cities (RR = 1.02; 95% CI = 1.01–1.04) was associated with higher CVD mortality. No clear associations were observed for sub-city greenness, population density, and the presence of mass transport infrastructure in cities.

**Conclusions:**

Our findings emphasise the impact of urban inequalities on cardiovascular health, while also identifying education as an important and modifiable urban environmental feature that could be amenable to public health policies aimed at addressing disparities in CVD mortality within Latin American cities.

Non-communicable diseases (NCDs) comprise 71% of all deaths worldwide, resulting in the annual death of 41 million individuals [1]. However, these deaths, especially those related to cardiovascular diseases (CVDs) and their major risk factors, are not uniformly distributed worldwide [2]. While CVD mortality rates have been decreasing in high-income countries (HICs), they have been increasing in low- and middle-income countries [2], where currently 77% of all NCD deaths occur [1]. In Latin America, CVDs have historically been among the leading causes of NCD-related mortality, responsible for approximately 27% of all deaths in the region in 2019 [3]. Nevertheless, mortality rates and associated risk factors, particularly those related to NCDs and CVDs, exhibit significant variation across cities in Latin America [3–6].

NCDs, such as CVD, are the combined result of environmental, behavioural, and stress-related risk factors [1]. Notably, city environmental factors play a crucial role in determining the distribution of CVD deaths within countries [1,5,6], as they can influence both behavioural and stress-related factors among their inhabitants [7–11]. For example, cities with higher landscape fragmentation [7], lack of green space [7,8], crowded settlements [9], and worse living conditions [9,10] are associated with higher psychosocial stress among their residents in HICs; while the availability of recreational areas [11], green spaces [7,8], and lower population density [12] in cities have been associated with higher physical activity. In addition, it has been shown that worse socioeconomic conditions (*i.e.* educational level [13], income level [14], and occupational class [15]), higher street connectivity, and road proximity [16] are associated with higher CVD mortality. Other city environmental factors, such as air pollution, can also have a direct impact on CVD mortality [17]. Therefore, a growing body of evidence in HICs supports understanding of the role of urban environments in shaping CVD mortality [3,16]. However, specific associations between built and social environments and CVD mortality across and within cities in low- and middle-income countries are still understudied.

The urbanisation process has been spreading worldwide, accompanied by an unfair distribution of health and wealth among individuals in many urban centres [18]. For instance, >70% of the Latin American and Caribbean population resides in urban areas [19] with highly diverse mortality profiles [5], distinct social and built environments, as well as considerable heterogeneity in housing, urbanisation, socioeconomic status [20–22], and access to healthcare [23]. These factors might explain differences in the distribution of CVD deaths [3] between and within Latin American cities. Investigating the association between modifiable urban environmental features and CVD mortality in the Latin American region is relevant to public health, particularly to identify protective urban health factors across and within cities, to guide the implementation of public policies aimed at reducing CVD mortality. Thus, we aim to characterise the variability in CVD mortality across and within cities within and between Latin American countries, and to explore the associations between social and built environment characteristics and CVD mortality between and within Latin American cities and countries.

## METHODS

### Study setting

We used data from the *Salud Urbana en América Latina* (SALURBAL) study [20,24], which has compiled and harmonised information on mortality, health, population, and built and social environment for Latin American cities with a population of ≥100 000 inhabitants in 2010. We defined the cities as administrative units or clusters of adjacent administrative units (*i.e.* sub-cities) that encompass the built-up area of urban agglomerations, as determined by satellite imagery. Each city is composed of sub-city units (*e.g.* municipalities, departamentos, or comunas). We used data from 342 cities and 1252 sub-city units in nine countries (Panama, Chile, Argentina, Costa Rica, Mexico, Brazil, Colombia, El Salvador, and Guatemala) for which all variables of interest were available (Table S1 in the **Online Supplementary Document**). Due to the limited number of cities in certain countries in Central America, we combined all countries in the Central American region (*i.e.* Panama, Costa Rica, El Salvador, and Guatemala) in some analyses (Table S2 in the **Online Supplementary Document**). The detailed sampling procedure and data harmonisation methods employed in the SALURBAL study can be found elsewhere [24].

### CVDs mortality data

We compiled mortality data for 2019 from vital registration systems as available in each country, except for El Salvador, which only had information for 2018 (Table S1 in the **Online Supplementary Document**) [24]. Mortality records included information on sex, age at death, sub-city of residence at the time of death, and cause of death. CVD deaths were defined as records with International Classification of Diseases-10 codes I00–99, which correspond to the CVD category in the World Health Organization Global Health Estimates classification. We imputed missing information on the age and sex of the decedent, which accounted for <1%, through conditional probabilistic imputation for each country and year using age, sex, cause of death, year, and country. Specifically, we probabilistically imputed missing age or sex to an age group or sex category, based on the observed distributions of these variables within their respective cause of death categories. Similarly, we probabilistically imputed missing cause of death information to either ill-defined diseases or injuries of ill-defined intent, based on age and sex [24]. We proportionally redistributed mortality records coded as ill-defined disease or injury using age, sex, country, and year [5]. We addressed the incomplete coverage of all deaths by calculating correction factors using an ensemble of death distribution methods by city [5]. Finally, we restricted CVD deaths to decedents aged ≥20 years and aggregated them by age groups (20–29, 30–59, 60–79, and ≥80 years) and sex for each sub-city unit. We obtained population denominators from national census bureaus, including counts of people by sub-city, year, age, and sex [5].

### Urban feature predictors

We evaluated urban features available in the SALURBAL study database [24] that represented dimensions of the city that are potentially amenable to urban policies, including demographic, built, and social environmental features.

#### Demographic data

We used sub-city demographic information, including total population and population density (*i.e.* total population / built-up area), which represents the population per km^2^ within the built-up area of each sub-city unit [24]. We obtained the built-up area data from the Global Urban Footprint data set derived from TerraSAR-X and TanDEM-X images [24]. We assessed population density at the sub-city level, as it may vary within cities [25].

#### Built environment data

We examined built environment indicators at the city and sub-city levels. At the city level, we considered urban patch density – the number of urban patches per 100 ha – and the percentage of urban area, both derived from the Global Urban Footprint dataset using TerraSAR-X and TanDEM-X images, which together reflect the degree of landscape fragmentation [24]. At the sub-city level, we focused on street connectivity, measured using OpenstreetMap and OSMNx data. Specifically, we used street intersection density, defined as the node density of all nodes with more than one street connected to them [24]. We explored street connectivity at the sub-city level as it may affect residents differently depending on where they live in the city [25]. We analysed landscape fragmentation at the city level as it may impact all residents regardless of where they live in the city [25]. We standardised all built environment features to have a mean of 0 and a standard deviation (SD) of 1 (*i.e.* Z-score).

#### Natural environment data

We explored the natural environment within cities as greenness, measured as the median (MD) of annual maximum normalised difference vegetation index at the sub-city unit level. We gathered the data from Moderate Resolution Imaging Spectroradiometer satellite (product code: MOD13Q1 V6), which measures the combined effect of vegetation cover, biomass, and photosynthetic activities on a −1 to 1 scale, with values closer to 1 indicating a stronger presence of vegetation [26]. We explored the normalised difference vegetation index at the sub-city level, as it may affect residents differently depending on where they live in the city [25].

#### Mass transport infrastructure data

We created a city-level variable for mass transport infrastructure, indicating the presence or absence of subway or bus rapid transit (based on BRTData, OpenstreetMap, and local resources) [24]. We measured it at the city level because such infrastructure can influence transportation behaviours across the entire city [25].

#### Social environment data

We characterised the social environment using a social environment index score (SES), which combines the proportions of the population aged >25 years who have completed secondary and university education [27], based on educational attainment at the sub-city level. We standardised these variables to Z-scores and summed to create the educational attainment SES. Thus, higher levels of the SES serve as a proxy for higher levels of educational attainment. We chose to evaluate the educational attainment variable as a proxy of socioeconomic position because it has previously been shown to be associated with a lifetime risk of CVD, regardless of other important socioeconomic characteristics [28]. Given the heterogeneity in educational attainment within Latin American cities [5], we analysed this variable at the sub-city level to capture the within-city variations.

### Statistical analysis

#### Descriptive analysis

We calculated age-adjusted CVD death rates for all sub-cities using the World Health Organization 2000–25 standard population as the reference, and examined their distribution along with that of potential predictors.

#### Modelling

We applied multilevel models to account for the hierarchical structure of the data, accommodating nested dependencies and variance across sub-city, city, and country levels. To account for the overdispersion observed in the distribution of CVD death counts, we used a negative binomial distribution. We used two distinct model approaches to estimate the associations between social and built environmental features and CVD death counts. First, we used four-level negative binomial models with sub-city units nested within cities and countries (random intercepts for countries, cities, and sub-cities). Second, to estimate these associations for each country individually, we used three-level negative binomial models of sub-city units nested within cities (random intercepts for cities and sub-cities). In both approaches, we included the CVD death counts – at the sub-city level – as a dependent variable, SES and built environment indicators (Z-score) as independent variables, age and sex for adjustment, and the total population adjusted for undercounting (*i.e.* total population × estimated registration coverage) as an offset term. We examined each social and built environment predictor in separate models (*i.e.* single exposure models). Subsequently, we included all built and social environment predictors in the same model (*i.e.* multiple exposure model). The correlations between covariates were weak to moderate (Table S4 in the **Online Supplementary Document**). Additionally, to facilitate interpretation, we also adjusted models that included landscape fragmentation for the percentage of urban areas in the unit. We interpreted the exponentiated coefficients of interest as the rate ratio (RR) of CVD mortality per 1-SD increase in the predictor of interest, adjusted for age and sex.

To decompose the variability of CVD mortality between each level (*i.e.* country, city, and sub-city level), we used generalised linear mixed models with CVD death rates as the dependent variable with no predictor on a three-level linear mixed model with random intercepts for country, city, and sub-city units. We estimated the proportion of variability at each level by dividing the variance of each level’s random intercept by the model's total variance. We applied this approach to both crude and age-adjusted CVD death rates at the sub-city level, both of which were normally distributed.

We fitted all models using *R*, version 4.3.1 (R Core Team, Vienna, Austria) and the glmmTMB package.

## RESULTS

### Sample characteristics

We included 1252 sub-cities nested in 342 cities and nine countries. About 34% of sub-city inhabitants aged >25 years had completed secondary education, and 9% had completed university education. Across all cities, we calculated the normalised difference vegetation index (MD = 0.79; interquartile range (IQR) = 0.67–0.85), patch density (MD = 0.57; IQR = 0.38–0.82), street intersection density (MD = 7.92; IQR = 3.10–23.10), population density (MD = 5179; IQR = 3912–7531), and mass transport infrastructure (n = 50; 14.62%) (**Table 1**). We also examined the environmental feature distributions by country (Figures S1–5 in the **Online Supplementary Document**).

**Table 1:** Sample characteristics for Latin American sub-city and city units*

	Overall	Central America†	Chile	Argentina	Mexico	Brazil	Colombia
**Sub-cities, n**	1252	558	81	108	405	422	83
**Cities, n**	342	102	21	32	92	152	35
**% of people with university education or above over 25 years‡**	8.90 (5.35–13.73)	12.70 (7.64–22.31)	4.49 (2.54–6.76)	9.10 (5.75–12.39)	8.68 (5.34–13.79)	8.91 (5.47–13.25)	9.10 (5.94–12.89)
**% of people with secondary education or above over 25 years‡**	34.34 (26.02–41.57)	40.01 (29.03–51.61)	39.45 (34.44–48.18)	36.37 (29.58–41.50)	26.93 (19.38–35.08)	36.09 (30.10–41.58)	34.53 (30.10–40.84)
**Greenness, in Z-scores‡**	0.79 (0.67–0.85)	0.85 (0.73–0.88)	0.53 (0.24–0.73)	0.64 (0.36–0.79)	0.73 (0.63–0.82)	0.82 (0.78–0.86)	0.84 (0.81–0.87)
**Landscape fragmentation, in patches/km^2^§**	0.57 (0.38–0.82)	0.71 (0.53–0.93)	0.44 (0.28–0.44)	0.76 (0.18–0.76)	0.55 (0.39–0.69)	0.65 (0.35–0.91)	0.43 (0.14–0.57)
**Street connectivity, in intersections/km^2^‡**	7.92 (3.10–23.10)	12.79 (3.11–46.36)	23.50 (4.49–100.70)	13.09 (2.43–73.26)	6.03 (2.60–18.08)	8.04 (3.85–18.59)	5.13 (1.85–10.61)
**Population density, in 1000 inhabitants/km^2^‡**	5179 (3912–7531)	5008 (3722–7263)	4487 (3518–6044)	6401 (4266–9749)	7043 (5101–11 022)	5019 (3975–6693)	10 536 (7057–13 665)
**Presence of mass transport infrastructure, n (%)§**	50 (14.62)	3 (30.00)	3 (14.29)	3 (9.38)	8 (8.70)	26 (17.11)	7 (20.00)
**CVD death rate, in deaths/100 000 inhabitants‡**	230 (194–267)	210 (177–256)	164 (145–181)	250 (212–275)	235 (200–269)	241 (208–277)	207 (181–238)

CVD – cardiovascular disease

*Values presented as median (interquartile range) unless specified otherwise. Countries are arranged in descending order based on their GDP *per capita*.

†Central America – Panama, Costa Rica, El Salvador, and Guatemala.

‡Sub-city level.

§City level.

**Figure 1:**
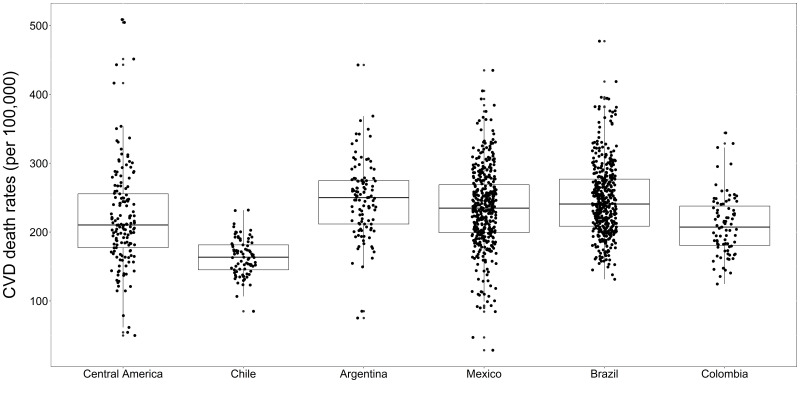
Variability in age-adjusted CVD death rates in 1252 sub-cities of 342 Latin American cities by country. Central line represents the median (50th percentile) sub-city expected CVD death; box limits represent the 25th and 75th percentiles. Central America – Panama, Costa Rica, El Salvador, and Guatemala. CVD – cardiovascular disease.

We observed heterogeneous age-adjusted CVD death rates across sub-cities within and between Latin American countries (**Figure 1**), with greater age-adjusted CVD death rate variability observed between sub-cities (60%) than cities (21%) and countries (19%) (Table S3 in the **Online Supplementary Document**). Argentina had the highest age-adjusted CVD deaths per 100 000 inhabitants (MD = 250), followed by Brazil (MD = 241), Mexico (MD = 235), Central America (MD = 210), Colombia (MD = 207), and Chile (MD = 164) (**Table 1**). Finally, the highest IQR in CVD death rates across sub-cities was observed for Central America (IQR = 177–256), followed by Brazil (IQR = 208–277), Argentina (IQR = 212–275), Colombia (IQR = 181–238), and Chile (IQR = 145–181).

### Predictors of CVD mortality in Latin American sub-cities

In single exposure models, every additional SD increase in educational attainment was associated with a 4% lower CVD death rate in all countries combined (RR = 0.96; 95% confidence interval (CI) = 0.95–0.97) (**Table 2**). In country specific analyses, a one SD higher value of educational attainment was associated with lower CVD rates in Central America (RR = 0.93; 95% CI = 0.89–0.96), Chile (RR = 0.90; 95% CI = 0.88–0.93), Argentina (RR = 0.94; 95% CI = 0.92–0.96), Brazil (RR = 0.92; 95% CI = 0.91–0.93), and a higher CVD rate in Mexico (RR = 1.03; 95% CI = 1.01–1.04), with no association in Colombia. Greenness was not associated with CVD mortality in the overall or the country-specific analyses, but was associated with a lower CVD rate in Mexico (RR = 0.97, 95% CI = 0.95–0.98). Higher fragmentation was not associated with CVD mortality in the overall pooled country analysis. However, living in cities with higher landscape fragmentation was associated with lower CVD mortality in Central America (RR = 0.92; 95% CI = 0.86–0.98) and Mexico (RR = 0.96; 95% CI = 0.92–0.99). Street connectivity demonstrated no association with CVD mortality in the overall analysis. Still, living in sub-cities with higher street connectivity was associated with higher CVD mortality in Chile (RR = 1.05; 95% CI = 1.01–1.09) and Mexico (RR = 1.03; 95% CI = 1.01–1.04), and lower CVD mortality in Brazil (RR = 0.97; 95% CI = 0.96–0.99). Higher population density was not associated with CVD mortality in the overall analysis. However, living in sub-cities with higher population density was associated with higher CVD mortality in Mexico (RR = 1.04; 95% CI = 1.02–1.06) and lower CVD mortality in Argentina (RR = 0.96; 95% CI = 0.93–0.98). The presence of mass transport infrastructure was not associated with CVD mortality in the overall analysis, but was associated with lower CVD mortality in Colombia (RR = 0.85; 95% CI = 0.77–0.94). We also analysed these associations without combining all countries in Central America (Table S5 in the **Online Supplementary Document**).

**Table 2:** CVD mortality associated with a one SD higher value of city and sub-city characteristics*

		Population educational attainment†			Greenness†			Landscape fragmentation‡			Street connectivity†			Population density†			Presence of mass transport infrastructure‡	
	**RR (95% CI)**		***P*-value**	**RR (95% CI)**		***P*-value**	**RR (95% CI)**		***P*-value**	**RR (95% CI)**		***P*-value**	**RR (95% CI)**		***P*-value**	**RR (95% CI)**		***P*-value**
**Single exposure models§**																		
Overall	0.96 (0.95–0.97)		<0.05	1.00 (0.99–1.01)			0.98 (0.95–1.00)			1.00 (0.99–1.01)			1.01 (1.00–1.02)			0.99 (0.94–1.03)		
Central America‖	0.93 (0.89–0.96)		<0.05	1.01 (0.97–1.06)			0.92 (0.86–0.98)		<0.05	0.98 (0.94–1.02)			1.00 (0.96–1.05)			1.06 (0.86–1.31)		
Chile	0.90 (0.88–0.93)		<0.05	0.98 (0.94–1.02)			0.99 (0.95–1.03)			1.05 (1.01–1.09)		<0.05	1.03 (1.00–1.07)			1.00 (0.93–1.08)		
Argentina	0.94 (0.92–0.96)		<0.05	1.03 (0.99–1.06)			1.08 (0.88–1.33)			1.00 (0.96–1.03)			0.96 (0.93–0.98)		<0.05	1.19 (0.93–1.53)		
Mexico	1.03 (1.01–1.04)		<0.05	0.97 (0.95–0.98)		<0.05	0.96 (0.92–0.99)		<0.05	1.03 (1.01–1.04)		<0.05	1.04 (1.02–1.06)		<0.05	0.99 (0.90–1.08)		
Brazil	0.92 (0.91–0.93)		<0.05	1.02 (1.00–1.04)			0.97 (0.93–1.01)			0.97 (0.96–0.99)		<0.05	0.98 (0.96–1.00)			1.00 (0.94–1.06)		
Colombia	1.02 (0.98–1.06)			1.02 (0.97–1.06)			0.99 (0.92–1.06)			1.01 (0.97–1.05)			1.03 (0.99–1.08)			0.85 (0.77–0.94)		<0.05
**Multiple exposure models§**																		
Overall	0.95 (0.94–0.96)		<0.05	1.01 (0.99–1.02)			0.97 (0.95–0.99)		<0.05	1.02 (1.01–1.04)		<0.05	1.01 1.00–1.02()			0.98 (0.92–1.03)		
Central America‖	0.90 (0.85–0.95)		<0.05	0.91 (0.83–0.99)		<0.05	0.89 (0.82–0.97)		<0.05	0.95 (0.86–1.05)			0.96 (0.91–1.02)			0.93 (0.75–1.16)		
Chile	0.91 (0.88–0.93)		<0.05	1.03 (0.98–1.08)			0.96 (0.93–1.00)			1.06 (1.00–1.12)		<0.05	1.02 (0.98–1.07)			0.92 (0.82–1.03)		
Argentina	0.94 (0.91–0.97)		<0.05	0.99 (0.94–1.04)			1.07 (0.85–1.33)			1.03 (0.98–1.07)			0.98 (0.94–1.01)			0.96 (0.68–1.37)		
Mexico	1.01 (0.99–1.03)			0.98 (0.95–1.00)			0.98 (0.94–1.02)			0.99 (0.96–1.02)			1.03 (1.01–1.06)		<0.05	1.07 (0.97–1.18)		
Brazil	0.92 (0.91–0.94)		<0.05	0.99 (0.97–1.02)			0.96 (0.92–1.01)			0.99 (0.96–1.02)			1.01 (0.99–1.02)			0.95 (0.89–1.02)		
Colombia	1.01 (0.96–1.05)			1.03 (0.97–1.09)			1.00 (0.93–1.07)			1.03 (0.96–1.11)			1.02 (0.97–1.09)			0.83 (0.71–0.97)		<0.05

CI – confidence interval, CVD – cardiovascular disease, RR – rate ratio, SD – standard deviation *Countries are arranged in descending order based on their GDP *per capita*.

†Sub-city level.

‡City level.

§Single-exposure models include only one environmental feature, while multiple-exposure models include all environmental features. Both models are adjusted for age, sex, and total population as an offset term, with landscape fragmentation adjusted for the percentage of urban area.

‖Central America – Panama, Costa Rica, El Salvador, and Guatemala.

In multiple exposure models, higher levels of educational attainment remained associated with lower CVD mortality in overall countries analysis (RR = 0.95; 95% CI = 0.94–0.96) as well as in Central America (RR = 0.90; 95% CI = 0.85–0.95), Chile (RR = 0.91; 95% CI = 0.88–0.93), Argentina (RR = 0.94; 95% CI = 0.91–0.97), and Brazil (RR = 0.92; 95% CI = 0.91–0.94). Living in sub-cities with higher levels of educational attainment was no longer associated with higher CVD mortality in Mexico. Greenness was not significantly associated with CVD mortality in the overall countries analysis, but living in sub-cities with higher greenness was associated with lower CVD mortality in Central America (RR = 0.91; 95% CI = 0.83–0.99). Higher landscape fragmentation was associated with lower CVD mortality in all countries combined (RR = 0.97; 95% CI = 0.95–0.99), and specifically in Central America (RR = 0.89; 95% CI = 0.82–0.97). Higher street connectivity was associated with higher CVD mortality in the overall country analysis (RR = 1.02; 95% CI = 1.01–1.04). Higher population density remained unassociated with CVD mortality in the overall country analysis, while it was still associated with higher CVD mortality in Mexico (RR = 1.03; 95% CI = 1.01–1.06). Finally, the presence of mass transport infrastructure in cities was still not associated with CVD mortality in the overall analysis, but remained associated with lower CVD mortality in Colombia (RR = 0.83; 95% CI = 0.71–0.97).

## DISCUSSION

We evaluated the associations between built and social environments and CVD mortality and described its variability between 1252 sub-cities, 342 cities, and nine Latin American countries. We found greater variability in CVD death rates within cities as compared to between cities or countries. Some urban environmental features were associated with CVD mortality, but associations differed across Latin American countries. Specifically, sub-cities with higher educational attainment and cities with higher landscape fragmentation exhibited lower rates of CVD mortality in the overall country analysis, with varying magnitudes of association between countries. In contrast, cities with higher street connectivity exhibited higher rates of CVD mortality in the overall country analysis. Finally, sub-city greenness, population density, and the presence of mass transport infrastructure in cities showed no clear associations with CVD mortality. Overall, our findings highlight the complex relationship between urban environmental features and CVD mortality in Latin America.

Few studies have explored CVD mortality and its urban predictors across Latin American cities. Our results not only reaffirm the existing understanding of heterogeneous mortality rates across Latin American cities and countries [5] but also extend this knowledge to the specific distribution of CVD mortality and its urban predictors across smaller areas within cities. Previous research has shown that higher city education was associated with lower city proportional communicable disease mortality [5]. However, they analysed proportionate mortality, a different metric that may yield distinct results. We build on this finding by investigating CVD cause-specific mortality, examining sub-city areas, and incorporating additional predictors. Our results also align with prior SALURBAL work showing that higher educational attainment at the sub-city level is associated with lower rates of hypertension [25] and CVD mortality [26] in Latin American countries.

The greater intra-city variability in CVD mortality, compared to inter-cities and inter-countries, highlights significant health inequalities within Latin American cities. The higher intra-city inequalities likely stem from historical disparities in resource distribution and social segregation during the urbanisation process in Latin American cities, affecting access to healthcare and education [18,21,22]. In contrast, inter-city and country differences are less pronounced, as the averages soften the observed internal variability.

Understanding the links between social conditions and CVD requires considering the stages of the epidemiological and nutritional transition. The lower CVD mortality rates observed among Latin American sub-cities with higher educational attainment are expected in countries in advanced stages of the epidemiological transition [29]. Countries at the fourth stage of the epidemiological transition experience a decline in age-specific mortality, which is accompanied by a shift of non-communicable burden to older ages with a relatively rapid improvement in survival concentrated among them [30]. This stage is marked by improved medical care and public health measures that benefit older age groups, reducing risk factors and NCD mortality [30]. Additionally, areas with lower levels of educational attainment are associated with poverty [31,32] and lower access to schools, healthcare facilities, and other health-related resources, including healthy foods, recreational facilities, and healthcare (geographical marginalisation) [33], resulting in higher CVD rates [30,34]. The nutritional transition is also important to consider, as dietary behaviours are important risk factors for CVD events [35]. In wealthier areas at later stages of the nutritional transition, residents have more access to resources to improve their dietary choices and physical activity through more local resources and supportive policies aimed at preventing obesity [36,37]. This could explain the lower CVD mortality observed in sub-cities with higher educational attainment in Panama, Chile, Argentina, Costa Rica, and Brazil, which are among the highest *per capita* gross domestic products in Latin America [38]. Nevertheless, it is important to consider that there are critical socioeconomic disparities in food access [39–41] and many other CVD risk factors within Latin American countries [5,39–42], which makes it hard to characterise Latin American countries in a specific stage of the epidemiological or nutritional transition, as multiple stages of the transition may coexist.

The higher CVD mortality rates observed among Mexican sub-cities with higher educational attainment are expected in countries in earlier stages of the epidemiological transition [29]. Countries at the third stage of the epidemiological transition are characterised by initial improvements in sanitisation, living standards, and medical and public health measures [29]. Consequently, inhabitants who would have died from infectious or parasitic diseases during earlier stages of the epidemiological transition survive longer and face the risk of dying from NCDs at older ages [29]. Moreover, individuals with higher education in earlier stages of nutritional and epidemiological transitions have higher incomes, greater access to high-calorie foods, and less time spent in physical activity, leading to obesity and obesity-related chronic diseases, such as CVD [36,37]. This may explain the findings for the sub-cities in Mexico, which are still in earlier stages of the nutritional transition [43]. Additionally, Mexico is a highly unequal country with many poor sub-cities within its territory [44], which may explain the lack of access to proper health technology and facilities, sanitisation, and infrastructure in many Mexican sub-cities [45,46]. Mexico’s health system in 2019 was characterised by limitations in access to health services, facilities, and high-cost treatments [47]. The limited access to healthcare, combined with the country’s current stage in the nutritional transition [43], could potentially explain the higher CVD mortality in higher social positions observed for 2019 in the region.

Higher greenness has been linked to beneficial effects for cardiovascular health in HICs [47–49], as well as for mental health [50] and physical activity [51], which also play essential roles in preventing CVD events [3]. However, very few studies have investigated the association between greenness and CVD events in Latin American countries compared to HICs [26,47–49]. A study conducted in Latin America found that cities with higher greenness and education levels experienced lower CVD mortality rates compared to cities with lower greenness and education levels [26]. We observed lower CVD mortality in sub-cities with higher greenness in Central America; however, there were no clear associations between sub-city greenness and CVD mortality across countries. The quality of green spaces, influenced by factors such as size, safety, and infrastructure, may impact the accessibility and usability in these areas [26,52], which we did not assess due to a lack of data. Not surprisingly, it has been found that higher SES groups have more access to green spaces than lower SES groups in HICs [49], but we still lack evidence in lower-income countries. Several hypotheses have been proposed to explain the associations between CVD and greenness, such as mitigation of harmful environmental exposures as heat, noise and air pollution; encouraging physical activity; facilitating social interactions and cohesion; improving mental health by reducing stress, anxiety and depression; and developing a healthy and robust immune system – hence a reduced risk of atherosclerosis – by exposure to microbial biodiversity of the natural environment [47]. Therefore, the absence of associations deserves further exploration with improved measures of greenness accessibility and usability [52].

Previous research in Latin American cities has shown a higher proportionate CVD/NCD mortality in cities with higher fragmentation or lower street connectivity [5]. Conversely, we found that residing in cities with higher city-level landscape fragmentation or lower sub-city street connectivity was associated with lower sub-city CVD mortality. Several factors could explain the observed differences. We defined CVD mortality at the sub-city rather than the city level, and adjustments involve a different set of variables compared to the previously reported city-level analyses. Additionally, while the previous research relied on proportionate mortality [5], we used death rates, indicating a difference in metrics and potentially yielding different results. Similar to our findings, a previous SALURBAL study in Latin American cities found that residing in sub-city areas with higher street-connectivity and cities with lower landscape fragmentation was associated with higher obesity [53], demonstrating consistency of our findings regarding CVD mortality and its correlated risk factors.

Higher population density and a lack of mass transit availability were previously associated with lower odds of having hypertension [25]. However, we found no clear association with population density or mass transit availability. The long causal chains and lags between built environment exposures and CVD mortality may explain some of these null findings. A previous SALURBAL study demonstrated that cities with higher landscape fragmentation are densely populated capital cities in Latin America with higher socioeconomic conditions that expanded their urban environment by developing their transport network, thus fragmenting their habitats into smaller patches with higher street connectivity [54]. Larger fragmented cities with a good transport infrastructure and accessible walkable streets may promote higher walkability [51,54], which is negatively associated with several CVD risk factors, including sedentary behaviours, obesity [52], hypertension, dyslipidemia, and diabetes [16,51]. However, the quality of infrastructure and safety are equally crucial to encourage physical activity in urban spaces [52,55], which we could not explore due to data limitations, potentially biasing our results. For instance, higher street connectivity is also associated with traffic congestion and elevated traffic-related air pollution [51], which is linked to hypertension risk [51]. This suggests that higher street intersection density, without considering the infrastructure quality, could indicate increased traffic rather than walkability. Therefore, the small effect associations we found should be interpreted with caution, as they may be influenced by unmeasured factors which could provide a more comprehensive understanding of the urban factors shaping CVD mortality.

To address disparities and enhance cardiovascular health in Latin America, social and health policy interventions and urban structural planning can improve cities’ urban environment and encourage healthier behaviours [50,55,56]. The built environment features we explored are related to cities' walkability [52,54,55], which is a key factor in promoting physical activity within cities [51,54], consequently enhancing cardiovascular health [3]. Improving cities' walkability and leisure physical activity can be achieved by enhancing the accessibility and usability of built urban environments [52,55] by providing parks, recreation centres, and walking or cycling trails separate from roads with secure parking for bicycles [57]. Finally, educational attainment could be improved by programmes and policies to support students and prevent school dropout [58], such as work (*e.g.* paid work experience or summer jobs conditioned on school attendance), training (*e.g.* educational and vocational training for employment purposes and support for disadvantaged school youth) [58], or income transfer programmes tied to educational attainment in local schools (*e.g. Bolsa Família* programme in Brazil) [59].

This study has several strengths. First, we analysed data on all 495 000 registered CVD deaths among 200 million residents of 1252 sub-cities within 342 cities from nine Latin American countries with heterogeneous social, economic, and built environmental features. Second, we used harmonised and standardised mortality records for each sub-city stratified by age and sex and corrected several limitations that are common in vital registration records analysis [24]. For instance, we addressed deaths coded as ‘ill-defined causes’ by redistributing them through conditional probabilistic imputation by age, sex, country, and year, and accounted for incomplete registration of deaths. Third, we categorised CVD deaths similar to previous studies [5,20] to improve the comparability of our findings. Lastly, our results are based on robust multilevel models exploring single and multiple exposure associations.

This study also has limitations. First, the method applied to estimate mortality coverage levels may under- or overestimate true mortality rates. However, a previous study has demonstrated that it performs relatively well in estimating mortality near its true values [60]. Additionally, prior studies have used this method [5,57,61], yielding reliable results consistent with the literature. Second, we used the cause of death information as coded in death records, which also included deaths coded as ‘ill-defined causes’. However, we addressed this limitation using proportional simple imputation by age, sex, country, and year [24]. Third, the reported age in both censuses and death records is commonly misreported, due to age heaping, overestimation in death records, and underestimation in population estimations [62]. To address this limitation, we aggregated our analyses across a large spectrum of age ranges. Furthermore, our association estimates reflect ecological correlations and cannot be extrapolated to individuals. However, they remain appropriate for drawing inferences at the city and sub-city levels [63]. Additionally, the cross-sectional nature of our study does not account for the long-term impacts of the social and built environment on CVD mortality. Moreover, we only examined the educational aspect of the social environment, which does not fully capture social inequalities. Other dimensions – such as housing, service provision, or income inequality [27] – may contribute to residual confounding and help explain the association between social environments and CVD mortality. Lastly, it is possible that environmental features at the neighbourhood level related to walkability or physical activity, such as street connectivity and greenness, may better explain CVD mortality within Latin American cities.

## CONCLUSIONS

We found considerable variability in CVD mortality across sub-cities both within and between Latin American countries. Sub-city education showed strong and consistent associations with CVD mortality, underscoring the role of social inequities. These findings highlight the importance of raising awareness about the health impact of social inequalities and the need for targeted public health policies within Latin American sub-cities to address disparities in CVD outcomes across social groups. Improving the urban environment and promoting healthier behaviours through social and health policy interventions may help reduce these disparities and enhance cardiovascular health in Latin America [50,55,56]. Given that CVDs remain the leading cause of death worldwide and contribute to rising healthcare costs [64,65], further consistent and comparable studies examining the complex relationship between the urban environment and CVD events are essential to guide policy at the global, national, regional, and sub-city levels.

## Additional material


Online Supplementary Document
