# Supplementary material for: Associations between social and built environments and cardiovascular disease mortality across 342 Latin American cities: results from the SALURBAL study
Source: J Glob Health. 2025 Oct 31;15:04262. doi: 10.7189/jogh.15.04262 (PMC12577666; doi:10.7189/jogh.15.04262)
Supplement: Online Supplementary Document [file jogh-15-04262-s001.pdf]

Supplement to: Oliveira DCRS, Gouveia N, Tumas N, Nazzal C, Alfaro T, Kroker-Lobos MF, Miranda JJ, Ramírez-Zea M, Oliveira TL, Caiaffa WT, Yamada G, Bilal U, Cardoso LO. Associations between social and built environments and cardiovascular disease mortality across 342 Latin American cities: results from the SALURBAL study. J Glob Health. 2025;15:04262.

Table S1. Data availability by year

Country*	Mortality	Social Environment	Built Environment
PA	2019	2010	2017
CL	2019	2002	2019
AR	2019	2010	2017
CR	2019	2011	2017
MX	2019	2010	2018
BR	2019	2010	2019
CO	2019	2005	2017
SV	2018	2007	2017
GT	2019	2018	2016

Footnote: Countries are arranged in descending order based on their per capita GDP.
*AR, Argentina; BR, Brazil; CL, Chile; CO, Colombia; CR, Costa Rica; GT, Guatemala; MX, Mexico; PA, Panama; SV, El Salvador.

Table S2. Number of sub-cities and cities information available by country

Country*	Sub-cities	Cities
PA	82	3
CL	81	21
AR	108	32
CR	29	1
MX	405	92
BR	422	152
CO	83	35
SV	22	3
GT	20	3
Total	1252	342

Footnote: Countries are arranged in descending order based on their per capita GDP.
*AR, Argentina; BR, Brazil; CL, Chile; CO, Colombia; CR, Costa Rica; GT, Guatemala; MX, Mexico; PA, Panama; SV, El Salvador.

Table S3. Proportion of variability in cardiovascular disease death rates between countries, cities and sub-cities

**Unit level**	**Crude variability (%)**	**Age-adjusted variability (%)**
Country	10%	19%
City	27%	21%
Sub-city	63%	60%

Footnote: % Variability between units is the proportion of variability of each cluster level in a multilevel linear regression of CVD death rates with no predictor, and random intercepts for country, city and sub-city.

Table S4. Correlation matrix between all city and sub-city environmental characteristics

	Educational attainment[a]	Greenness[a]	Landscape Fragmentation[b]	Percentage of urban area[b]	Street Connectivity[a]	Population density[a]	Presence of mass transport infrastructure[b]
Educational attainment[a]	1.00	-0.26	-0.04	0.08	0.36	0.03	-0.01
Greenness[a]	-0.26	1.00	-0.09	-0.33	-0.70	-0.15	-0.27
Landscape Fragmentation[b]	-0.04	-0.09	1.00	0.58	0.23	0.07	0.31
Percentage of urban area[b]	0.08	-0.33	0.58	1.00	0.47	0.51	0.69
Street Connectivity[a]	0.36	-0.70	0.23	0.47	1.00	0.31	0.38
Population density[b]	0.03	-0.15	0.07	0.51	0.31	1.00	0.45
Presence of mass transport infrastructure[b]	-0.01	-0.27	0.31	0.69	0.38	0.45	1.00

[a]sub-city level
[b]city level

Table S5. Rate ratios (95%) of cardiovascular disease mortality associated with a one SD higher value of city and sub-city characteristics for all cities by country adjusted for age and sex

Countries	Population educational attainment[a]		Greenness[a]		Landscape fragmentation[b]		Street connectivity[a]		Population Density[a]		Presence of mass transport infrastructure[b]	
	Ratio	CI	Ratio	CI	Ratio	CI	Ratio	CI	Ratio	CI	Ratio	CI
Single exposure models												
Panama	0.90*	0.84 – 0.96	0.99	0.92 – 1.08	0.88*	0.81 – 0.96	0.98	0.90 – 1.07	1.02	0.94 – 1.10	1.07	0.79 – 1.45
Chile	0.90*	0.88 – 0.93	0.98	0.94 – 1.02	0.99	0.95 – 1.03	1.05*	1.01 – 1.09	1.03	1.00 – 1.07	1.00	0.93 – 1.08
Argentina	0.94*	0.92 – 0.96	1.03	0.99 – 1.06	1.08	0.88 – 1.33	1.00	0.96 – 1.03	0.96*	0.93 – 0.98	1.19	0.93 – 1.53
Costa Rica	0.93*	0.88 – 0.98	1.04	0.99 – 1.09	NA	NA	0.97	0.92 – 1.01	1.02	0.97 – 1.08	NA	NA
Mexico	1.03*	1.01 – 1.04	0.97*	0.95 – 0.98	0.96*	0.92 – 0.99	1.03*	1.01 – 1.04	1.04*	1.02 – 1.06	0.99	0.90 – 1.08
Brazil	0.92*	0.91 – 0.93	1.02	1.00 – 1.04	0.97	0.93 – 1.01	0.97*	0.96 – 0.99	0.98	0.96 – 1.00	1.00	0.94 – 1.06
Colombia	1.02	0.98 – 1.06	1.02	0.97 – 1.06	0.99	0.92 – 1.06	1.01	0.97 – 1.05	1.03	0.99 – 1.08	0.85*	0.77 – 0.94
El Salvador	0.98	0.90 – 1.06	1.02	0.95 – 1.10	0.74	0.06 – 8.65	1.01	0.94 – 1.09	0.94	0.87 – 1.01	0.87	0.69 – 1.10
Guatemala	0.97	0.89 – 1.05	1.01	0.94 – 1.09	1.07	0.92 – 1.25	0.98	0.91 – 1.06	1.04	0.96 – 1.12	0.93	0.77 – 1.12
Multiple exposure models												
Panama	NA	NA	NA	NA	NA	NA	NA	NA	NA	NA	NA	NA
Chile	0.91*	0.88 – 0.93	1.03	0.98 – 1.08	0.96	0.93 – 1.00	1.06*	1.00 – 1.12	1.02	0.98 – 1.07	0.92	0.82 – 1.03
Argentina	0.94*	0.91 – 0.97	0.99	0.94 – 1.04	1.07	0.85 – 1.33	1.03	0.98 – 1.07	0.98	0.94 – 1.01	0.96	0.68 – 1.37
Costa Rica	NA	NA	NA	NA	NA	NA	NA	NA	NA	NA	NA	NA
Mexico	1.01	0.99 – 1.03	0.98	0.95 – 1.00	0.98	0.94 – 1.02	0.99	0.96 – 1.02	1.03*	1.01 – 1.06	1.07	0.97 – 1.18
Brazil	0.92*	0.91 – 0.94	0.99	0.97 – 1.02	0.96	0.92 – 1.01	0.99	0.96 – 1.02	1.01	0.99 – 1.02	0.95	0.89 – 1.02
Colombia	1.01	0.96 – 1.05	1.03	0.97 – 1.09	1.00	0.93 – 1.07	1.03	0.96 – 1.11	1.02	0.97 – 1.09	0.83*	0.71 – 0.97
El Salvador	NA	NA	NA	NA	NA	NA	NA	NA	NA	NA	NA	NA
Guatemala	NA	NA	NA	NA	NA	NA	NA	NA	NA	NA	NA	NA

Footnote: Countries are arranged in descending order based on their per capita GDP.

Single exposure models include only one environmental feature adjusted for age, sex, and total population as an offset term. Landscape fragmentation is additionally adjusted for the percentage of urban area in the unit.
Multiple exposure models include all environmental features adjusted for age, sex, and total population as an offset term. Landscape fragmentation is additionally adjusted for the percentage of urban area in the unit.
NA = Not available due to convergence issues caused by the small sample size, leading to estimate instability
[a]sub-city level
[b]city level
*P<0.05

Figure S1. Variability in educational attainment[a] in 1,252 sub-cities of 342 Latin American cities by country*

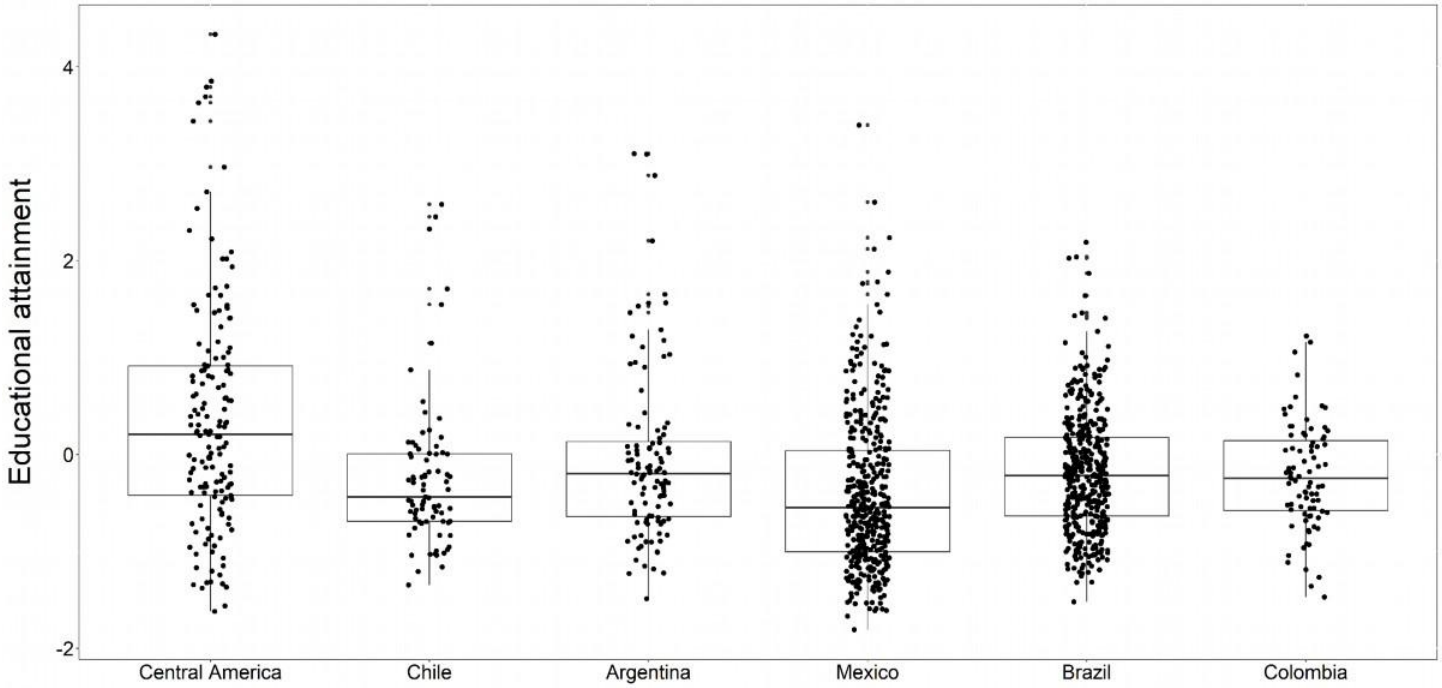

Footnote: Countries are arranged in descending order based on their per capita GDP.
[a]Educational attainment index score at the sub-city level

*Central line represents the median (50th percentile) sub-city educational attainment index score, box limits represent the 25th and 75th percentiles. Central America = Panama, Costa Rica, El Salvador, and Guatemala

Figure S2. Variability in greenness[a] in 1,252 sub-cities of 342 Latin American cities by country*
Footnote: Countries are arranged in descending order based on their per capita GDP.

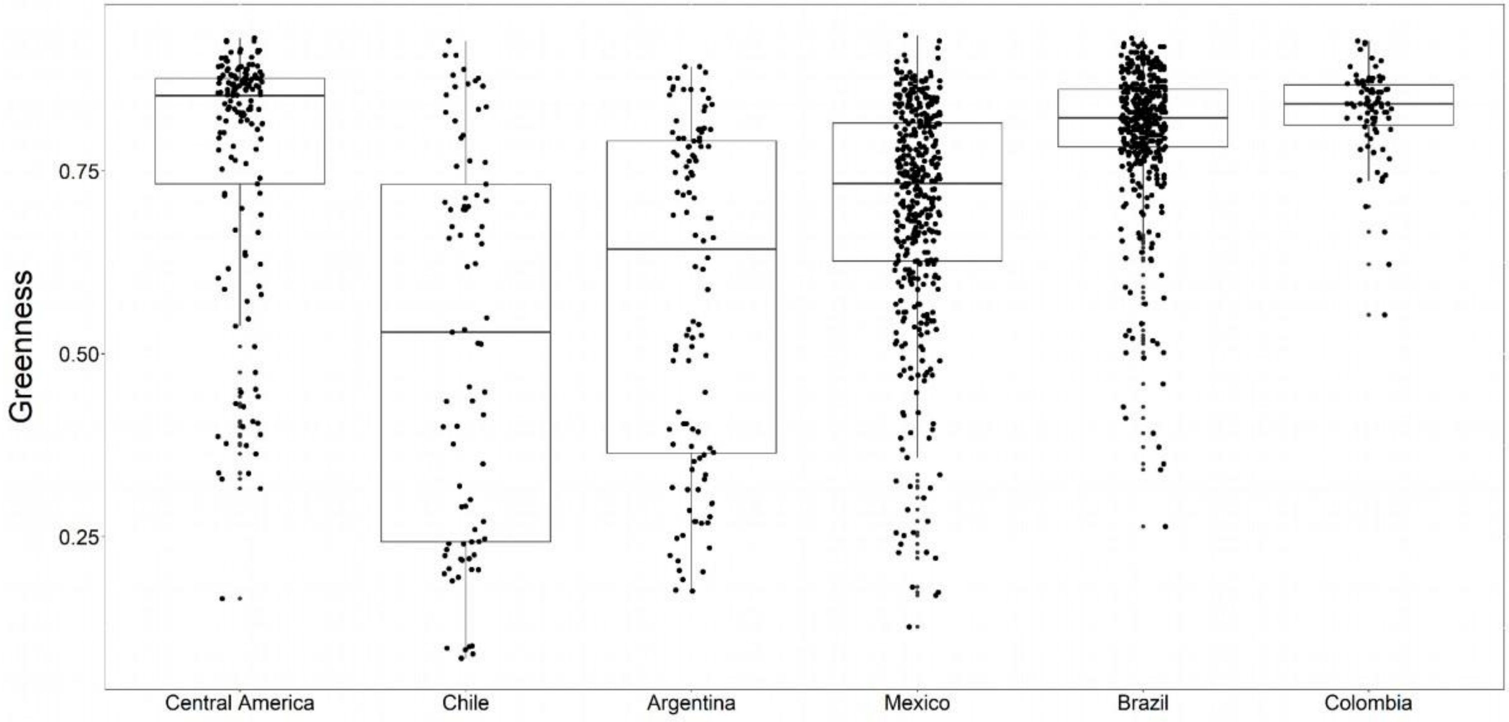

[a]Normalized Difference Vegetation Index at the sub-city level
*Central line represents the median (50th percentile) sub-city NDVI, box limits represent the 25th and 75th percentiles.
Central America = Panama, Costa Rica, El Salvador, and Guatemala

Figure S3. Variability in street connectivity[a] in 1,252 sub-cities of 342 Latin American cities by country*

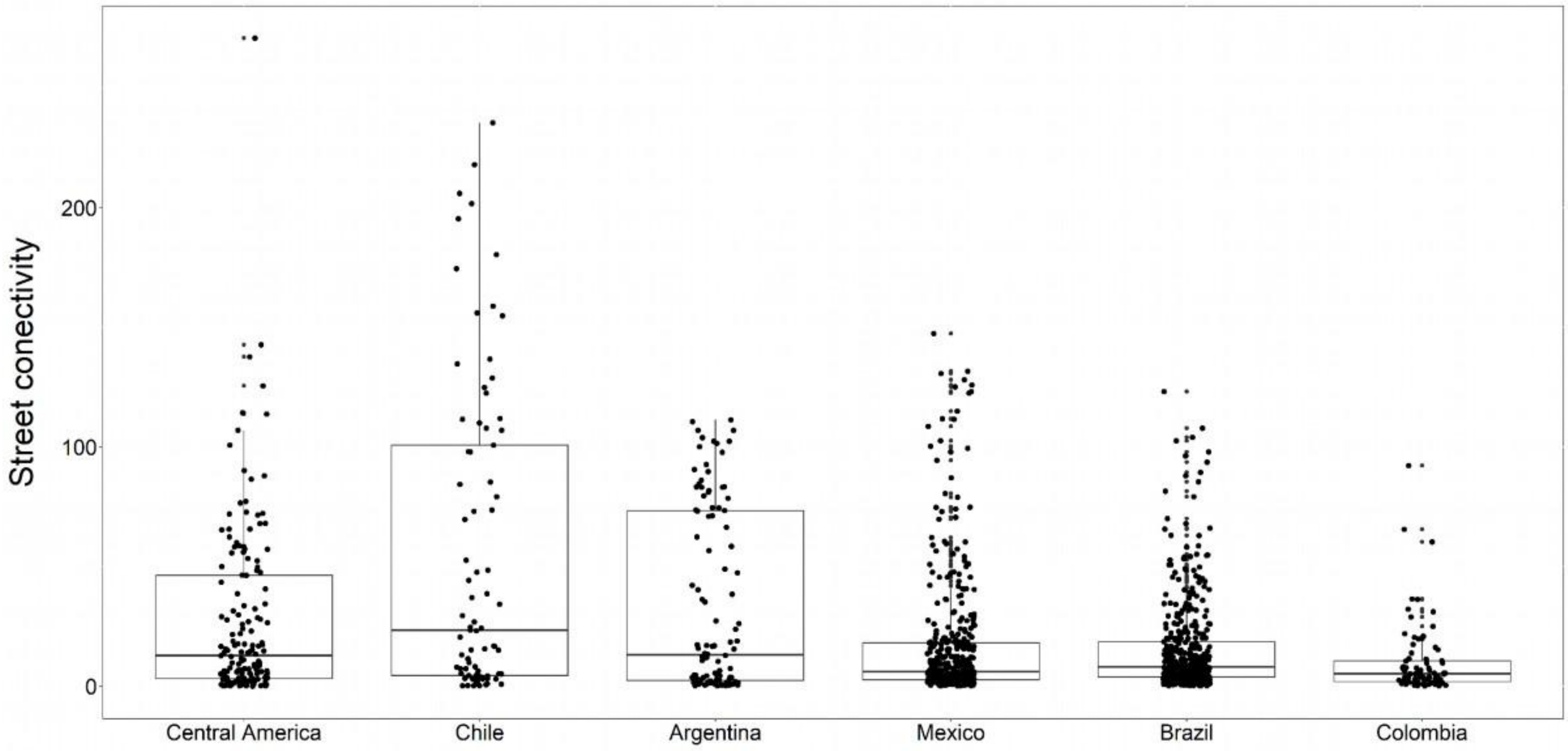

Footnote: Countries are arranged in descending order based on their per capita GDP.
[a]Street intersections per square kilometer at the sub-city level
*Central line represents the median (50th percentile) sub-city street intersection per square kilometer, box limits represent the 25th and 75th percentiles. Central America = Panama, Costa Rica, El Salvador, and Guatemala

Figure S4. Variability in population density[a] in 1,252 sub-cities of 342 Latin American cities by country*
Footnote: Countries are arranged in descending order based on their per capita GDP.
[a]1000

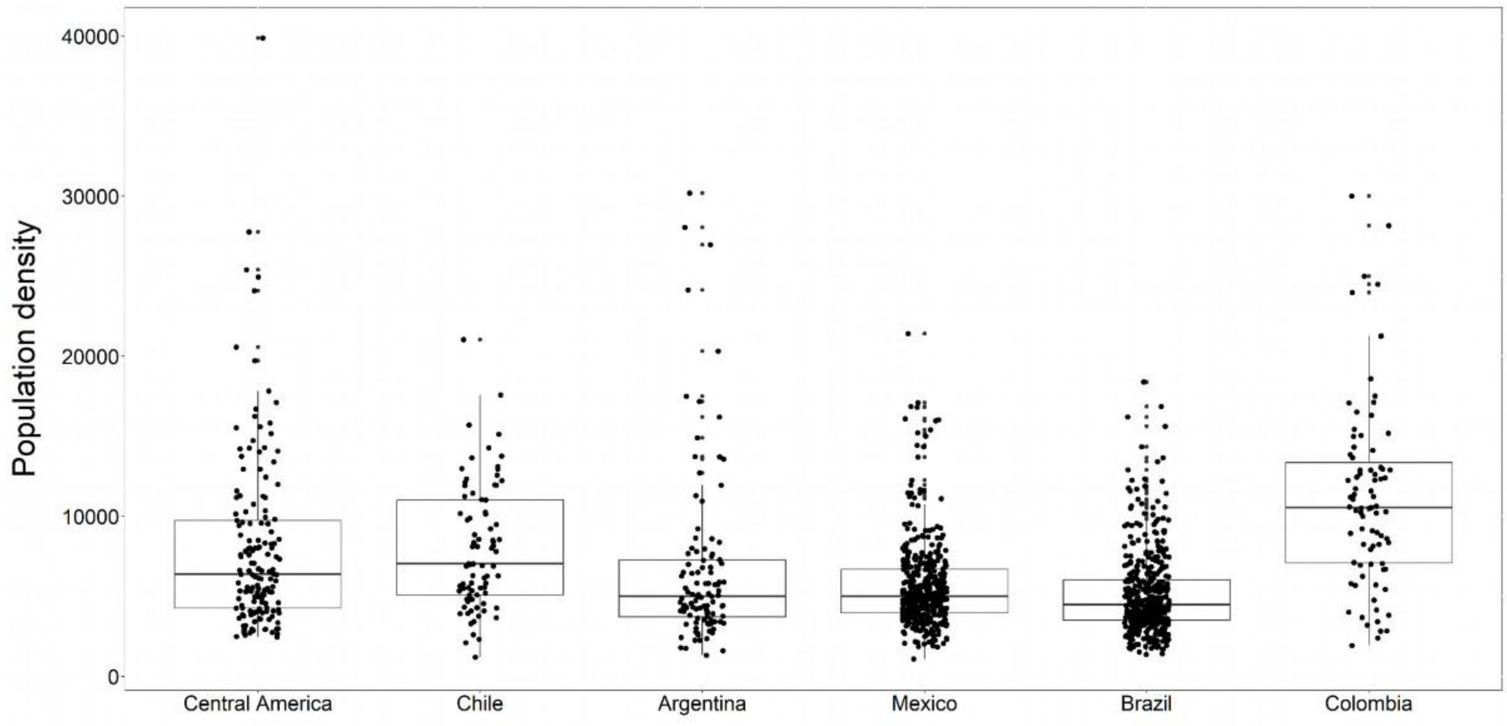

inhabitants per square kilometer at the sub-city level
*Central line represents the median (50th percentile) sub-city population density, box limits represent the 25th and 75th percentiles. Central America = Panama, Costa Rica, El Salvador, and Guatemala

Figure S5. Variability in landscape fragmentation[a] in 1,252 sub-cities of 342 Latin American cities by country*
Footnote: Countries are arranged in descending order based on their per capita GDP.

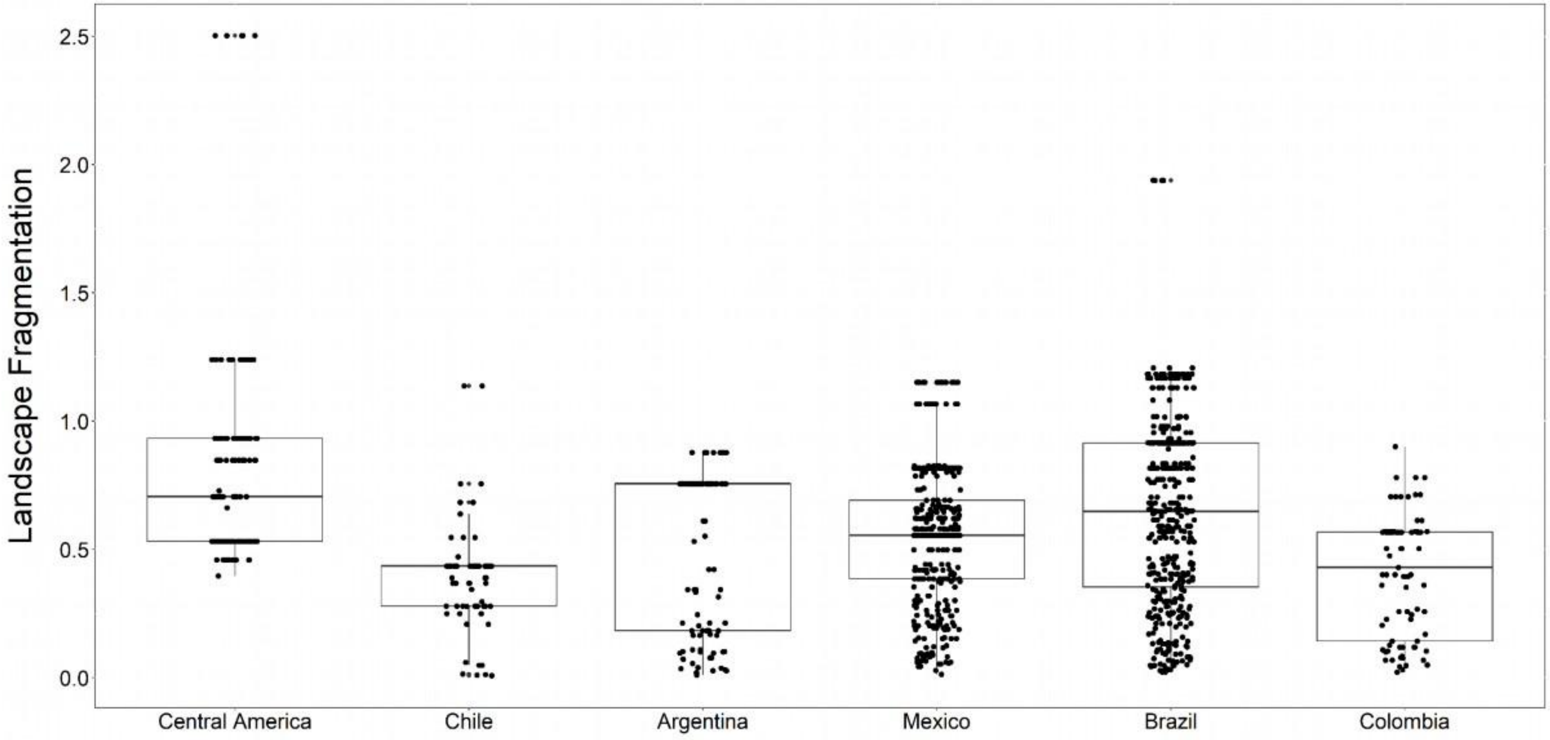

[a]Urban patches per square kilometer at the city level
*Central line represents the median (50th percentile) city landscape fragmentation, box limits represent the 25th and 75th percentiles. Central America = Panama, Costa Rica, El Salvador, and Guatemala.
